# Age and sex differences in human balance performance from 6-18 years of age: A systematic review and meta-analysis

**DOI:** 10.1371/journal.pone.0214434

**Published:** 2019-04-09

**Authors:** Simon Schedler, Rainer Kiss, Thomas Muehlbauer

**Affiliations:** 1 Division of Movement and Training Sciences/Biomechanics of Sport, University of Duisburg-Essen, Essen, Germany; 2 Department of Health and Social Affairs, FHM Bielefeld—University of Applied Sciences, Bielefeld, Germany; Universidade Federal da Bahia, BRAZIL

## Abstract

**Background:**

The process of growing leads to inter-individual differences in the timing of growth, maturational, and developmental processes during childhood and adolescence, also affecting balance performance in youth. However, differences in balance performance by age and sex in youth have not been systematically investigated yet.

**Objective:**

The objective of the present study was to characterize and quantify age- and sex-related differences in balance performance in healthy youth.

**Methods:**

A computerized systematic literature search was performed in the electronic databases PubMed, Web of Science, and SPORTDiscus. To be applicable for analysis, studies had to report at least one measure of static steady-state, dynamic steady-state, proactive or reactive balance in healthy children (6–12 years) and/or adolescents (13–18 years). Coding of the studies was done according to the following criteria: age, sex, and balance outcome. Study quality was assessed using the Appraisal tool for Cross-Sectional Studies. Weighted standardized mean differences were calculated and classified according to their magnitude.

**Results:**

Twenty-one studies examined age-related differences in balance performance. A large effect for measures of static steady-state balance (SMD_ba_ = 1.20) and small effects for proxies of dynamic steady-state (SMD_ba_ = 0.26) and proactive balance (SMD_ba_ = 0.28) were found; all in favor of adolescents. Twenty-five studies investigated sex-related differences in balance performance. A small-sized effect was observed for static steady-state balance (SMD_bs_ = 0.33) in favor of girls and for dynamic steady-state (SMD_bs_ -0.02) and proactive balance (SMD_bs_ = -0.15) in favor of boys. Due to a lack of studies, no analysis for measures of reactive balance was performed.

**Conclusions:**

Our systematic review and meta-analysis revealed better balance performances in adolescents compared to children, irrespective of the measure considered. Sex-related differences were inconsistent. These findings may have implications for example in terms of trainability of balance in youth that should be investigated in future studies.

## Introduction

Balance is generally referred to as the ability to keep the body’s center of mass within the base of support [1]. Integrating sensory information from visual, sensorimotor, and vestibular receptors and producing adequate muscle synergies, enables humans to achieve and maintain balance under static conditions where the base of support and the ground remain stationary (e.g., standing) as well as under dynamic conditions where the center of mass and the base of support shift (e.g., walking). According to Shumway-Cook and Woollacott [2], balance can further be subdivided into four types of balance hardly associated with each other: static steady-state balance (i.e., maintaining a steady position while standing/sitting), dynamic steady-state balance (i.e., maintaining a steady position while walking), proactive balance (i.e., anticipation of a predicted postural disturbance), and reactive balance (i.e., compensation of an unpredicted postural disturbance).

Depending on the measured variable, balance performance shows a U-shaped (e.g., postural sway) or inverted U-shaped (e.g, gait velocity) relationship with age across the lifespan illustrating increasing performance in youth, peak performance in young adults, and decreasing performance in seniors [3]. The development of balance performance throughout youth has been an area of particular research interest as it may assist in the early identification of diseases and/or disorders, designing training regimes, or understanding deteriorating balance performance in older adults and seniors. As indicated by for instance decreasing postural sway [4, 5] and increasing gait speed [6, 7], balance performance reportedly improves from early childhood onwards. The underlying developmental processes have been investigated in detail and it has been shown that physical factors (e.g., growth, weight gain) only marginally influence balance performance in youth [8, 9]. In fact, improved sensory integration [1, 10], the task-specific use of different postural control strategies [11, 12], and progressive brain maturation [13, 14] particularly account for improvements in balance performance in youth. However, there is still conflicting evidence whether these developments lead to differences in balance performance between school-aged children (6–12 years) and adolescents (13–18 years) in favor of the first or the latter. Some authors found adult-like blance performance in children at the end of the first decade of life (i.e., 7-10-year-olds [15], 11-13-year-olds [16]), indicating equal or even better balance performances of children compared to adolescents. Other studies [10, 17], however, reported better balance performances of adolescents compared to children. Hence, the true extent of age-related differences in balance performance in youth remains debatable.

Besides age-related differences in balance performance, research has also focused on sex-related differences. For example, in adults, better performances of females compared to males have been reported [18, 19], although some evidence suggests that these might be related to anthropometric differences [20]. Similarly, there is conflicting evidence with respect to sex-related differences in balance performance in youth. Several studies [17, 21, 22] reported better balance performance in girls compared to same-aged boys, which has been attributed to improved sensory integration [17], advanced neuromuscular development [21], and the use of more adult-like postural control strategies [22]. Furthermore, it has been suggested that compared to boys, girls are less hyperactive [10] and more attentive during balancing tasks [17]. However, there are also studies showing girls and boys to perform equally well in terms of balance performance [23, 24]. Consequently, it is still questionable, if and to what extent sex-related differences in balance performance in youth exist.

Although balance performance in youth has been studied extensively over the past decades, evidence regarding age- and sex-related differences is rather contradictory. Certainly, knowledge about the development of balance and maturation of involved systems in terms of differences between children and adolescents and between girls and boys may assist clinicians and practitioners in the early identification of developmental disorders and diseases or in the development of specific training regimes. Meta-analyses provide the highest level of evidence on the evidence pyramid [25] and, to the best of our knowledge, no study has systematically investigated age- and sex-related differences in balance performance in youth yet. Thus, the aim of this systematic literature review and meta-analysis was to characterize and quantify age- and sex-related differences in several variables of balance in healthy youth. We expected better balance performances in adolescents compared to children and in girls compared to boys.

## Methods

### Search of literature

A systematic literature search in the electronic databases PubMed, Web of Science, and SPORTDiscus was performed using the following Boolean search strategy: (("balance performance" OR "postural control" OR “postural balance” OR postural stability OR balance test OR sensory integration OR gait OR functional reach OR balance function OR balance ability) AND (children OR adolescent OR youth OR girls OR boys OR development OR maturation)). Additionally, the search was limited to publication date (1960/01/01-2018/08/31), age (6–18 years), English language, full-text original articles, and human species. Moreover, reference lists of included articles and relevant review articles were checked and analyzed to identify other studies potentially suitable for inclusion.

### Criteria for selection

Table 1 summarizes the applied selection criteria. To be eligible for inclusion, studies had to meet the following criteria: (a) participants were healthy; (b) participants were aged between six and 18 years; (c) at least one balance parameter was registered in the study; (d) results were reported either for children (6–12 years) and adolescents (13–18 years) or for girls and boys or for children and adolescents as well as for girls and boys. While most children start attending school at six years having achieved certain developmental and motor milestones (e.g., running, hopping) [26], pre-schoolers are even more heterogenous in terms of inter-individual development and were therefore excluded from this study. The other exclusion criteria were as follows: (a) participants were exclusively athletes, patients, and/or people with diseases; (b) reported data did not allow for calculation of effect sizes [27, 28]; (c) study authors did not reply to our inquiries to send original data [8, 29, 30]; (d) balance parameters were measured on an absolute scale (e.g., number of errors during single-leg stance); (e) balance performance was measured under dual- and/or supra-postural task conditions.

**Table 1:** Selection criteria.

Category	Inclusion criteria	Exclusion criteria
Population	healthy children (6–12 years) and/or adolescents (13–18 years)	patients, athletes
Measurement	balance during single-task	balance during dual- and/or supra-postural task
Outcome	at least one measure of static steady-state, dynamic steady-state, proactive, and/or reactive balance	reported data did not allow for calculation of effect sizes; author(s) did not respond to our inquiries
Study design	cross-sectional studies	intervention studies not reporting pre-intervention data for healthy controls matching the other inclusion criteria

Studies comparing balance performance of athletes, patients and/or people with diseases to that of healthy controls were included, if they reported relevant data for control group(s), which allowed for comparisons of balance performance between age groups and/or sex within the controls. Similarly, interventional studies were also included, if pre-intervention data of balance performance was reported, enabling us to analyze age- and/or sex-related differences. Study eligibility was assessed by two independent reviewers (SS, TM). A third vote (RK) was obtained, if the two reviewers disagreed about the eligibility of a study for inclusion. If studies reported results for age groups overlapping with our categories, we grouped them according to the predominating age. For example, Condon and Cremin [31] reported data for 12-15-year-olds, which we classified as data of adolescents as only the 12-year-olds would represent children, while the 13-, 14- and 15-year-olds represent adolescents.

### Study coding

Each included study was coded for the following criteria: number of participants, sex, and age. Due to the inconsistent findings on whether balance performance is adult-like in ten- to twelve-year-olds and according to previously established developmental models classifying adolescence into the age period of either 11.5–16 years [32], 12–18 years in girls and 14–18 years in boys [33], or 10–19 years in girls and 12–20 years in boys [34], the age-range of 6–12 years was defined for children, while 13-18-year-olds represent adolescents in our study. Balance performance was classified into the following categories, as suggested by Shumway-Cook and Woollacott [2]: static steady-state balance (i.e., maintaining a static position while standing), dynamic steady-state balance (i.e., maintaining a steady position while walking), proactive balance (i.e., anticipation of a predicted postural disturbance), and reactive balance (i.e., compensation of a postural disturbance). As some studies reported several variables within one outcome category, we prioritized the most commonly reported measure for each category to reduce heterogeneity between studies. Regarding static steady-state balance, highest priority was given to CoP displacements during single-leg stance with eyes open. Gait velocity during walking at preferred speed was used with reference to dynamic steady-state balance. In terms of proactive balance, highest relevance was given to the distance in the FR, while CoP displacements during perturbed single-leg stance were defined as most representative for reactive balance. If studies reported other measures as proxies of the aforementioned categories, alternative outcome(s) were used depending on the administered balance test and recorded parameter(s). Table 2 lists the preferred and alternative outcome(s) for each balance category.

**Table 2:** Preferred and alternative outcome(s). *BESS* Balance error scoring system, *CoP* Center of Pressure, *FR* Functional-Reach test, *LOS* Limits of stability *SOT* Sensory Organization Test, *TUG* Timed up and go test.

Category	Preferred outcome	Alternative outcome(s)
Static steady-state balance	CoP displacements during single-leg stance	time in balance SOT BESS Balance Index
Dynamic steady-state balance	gait speed during walking	cadence body sway body accelerations LOS
Proactive balance	reach distance during FR	TUG Star excursion balance test CoP displacements during self-perturbed stance Timed up and down stairs test
Reactive balance	CoP displacements during perturbed stance	-

### Study quality

The Appraisal tool for Cross-Sectional Studies [35] was used for quality assessment of included studies. It consists of 20 questions addressing study design, study quality, and risk of bias, which have to be answered with “yes”, “no”, or “do not know”. Seven questions refer to quality of reporting (1, 4, 10, 11, 12, 16, 18) and seven to study design (2, 3, 5, 8, 17, 19, 20). Another six questions (6, 7, 9, 13, 14, 15) relate to a possible risk of bias. Three questions (7, 13, 14) asking for potential non-responders were excluded from our analysis as this criterion was not applicable for the vast majority of included studies. Quality assessment was performed by two independent reviewers (SS, TM). If the respective reviewers did not reach a consensus, evaluation from a third expert (RK) was obtained.

### Statistical analyses

Age (i.e., children vs. adolescents) and sex (i.e., boys vs. girls) differences in balance performances were assessed using standardized mean difference (SMD), which is defined as follows:

$$SMD=\frac{Difference\;in\;mean\;outcome\;between\;groups}{Standard\;deviation\;of\;outcome\;among\;participants}$$

Two studies only reported confidence intervals (CI) [36] or standard errors (SE) [37] instead of standard deviations (SD). According to the Cochrane Handbook for Systematic Reviews of Interventions [38] we used the following formula to calculate SD from CI:

$$SD=\sqrt{N}\;x\;\frac{\left(upper\;limit-lower\;limit\right)}{t\;value}$$

where *N* is the number of subjects within a group, *upper* and *lower limit* the particular CI and *t value* is defined by the length of the CI and the degrees of freedom. To derive SD from SE we used the following formula [38]:

$$SD=SE\;x\;\sqrt{N}$$

where SE is the particular standard error and N is the number of subjects within a group. At first, SMDs and SDs were calculated for differences in balance performance by age group (i.e., children vs. adolescents) and sex (i.e., girls vs. boys) for comparisons within each study. Subsequently, computed SMDs and SDs were used to assess differences between components of balance (i.e., static/dynamic steady-state, proactive, and reactive balance) by age (SMD_ba_) and sex (SMD_bs_). Calculation of weighted mean SMD_ba_ and SMD_bs_ was done using a random-effects meta-analysis in Review Manager version 5.3. SMD_ba_ and SMD_bs_ can be negative or positive depending on the outcome measure. For example, less sway during a balance task would yield a negative SMD, whereas a longer time in balance would result in a positive SMD, although both represent good balance performance. Consistent with our hypotheses, positive SMD_ba_/SMD_bs_ indicate better balance performance of adolescents compared to children and of girls compared to boys, respectively. According to Cohen [39], values for SMD_ba_/SMD_bs_ of 0 ≤ 0.49 indicate small effects, values of 0.50 ≤ 0.79 indicate medium effects, and values of ≥ 0.80 indicate large effects. I^2^ and Chi^2^-statistics were used to assess heterogeneity between studies. In agreement with Deeks et al. [40], heterogeneity was classified as being either trivial (0% ≤ I^2^ ≤ 40%), moderate (30% ≤ I^2^ ≤ 60%), substantial (50% ≤ I^2^ ≤ 90%), or considerable (75% ≤ I^2^ ≤ 100%).

## Results

**Fig 1:**
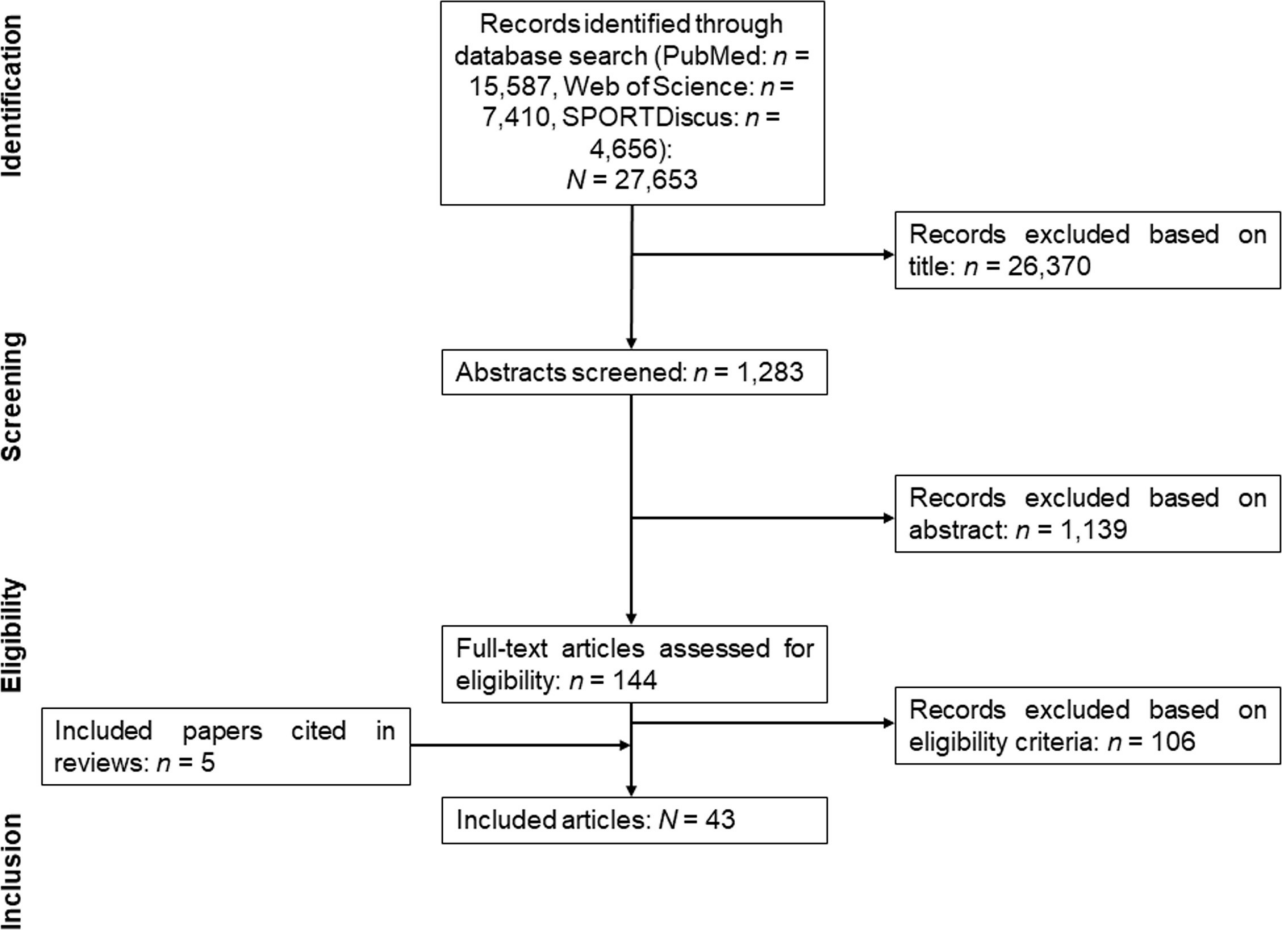
Flow chart illustrating the different phases of the search and selection.

Fig 1 illustrates the process of the systematic literature search. The systematic literature search revealed 27,653 studies potentially suitable for analysis. After screening titles, reading abstracts, and removing duplicates a total of 38 articles remained suitable for inclusion. Further five articles were identified from the reference lists of included articles and relevant reviews and found to be eligible for inclusion. Therefore, 43 studies were included in the final analysis. Eighteen studies reported data for balance performance of children and adolescents, 22 studies reported data for balance performance of boys and girls, and three studies reported data for both comparisons.

### Description of included studies

Table 3 shows the characteristics of the 21 included studies reporting measures of balance performance in children and adolescents. Twelve studies [5, 10, 17, 31, 41–48] reported variables of static steady-state balance, two studies [49, 50] analyzed outcomes of dynamic steady-state balance, and six studies [36, 37, 51–54] examined proxies of proactive balance. One study analyzed static and dynamic steady-state balance as well as reactive balance [55]. Data of a total of 9,716 subjects of whom 8,168 were categorized as children and 1,548 were grouped as adolescents were eligible for analysis. Age ranged from 6–13 years in children and from 12–19 years in adolescents.

**Table 3:** Included studies examining age differences (children vs. adolescents) in balance performance in youth.

References	No. of subjects; sex; age [years (range or mean ± SD)]; n	Balance test parameter; outcome	Results SMD_ba_ (95% CI)
An et al. [41]	39; F (15), M (24); 7–14	sSSB: one-legged stance, dominant leg, firm surface, eyes open; time in balance (60 s maximum)	7-9- vs. 12-14-year-olds: 0.92 (0.25, 1.59)
	7-9-year-olds; 19		
	12-14-year-olds; 20		
Barozzi et al. [42]	289; F (115), M (174); 6–14	sSSB: 52-s two-legged stance, barefoot, eyes open, firm surface; sway velocity (mm/s)	6- vs. 13-year-olds: 2.12 (1.43, 2.81)
			7- vs. 13-year-olds: 1.82 (1.29, 2.35)
	6-year-olds; 20		8- vs. 13-year-olds: 1.05 (0.56, 1.54)
	7-year-olds; 43		9- vs. 13-year-olds: 0.93 (0.40, 1.46)
	8-year-olds; 38		10- vs. 13-year-olds: 0.92 (0.45, 1.39)
	9-year-olds; 27		11- vs. 13-year-olds: 1.73 (1.14, 2.32)
	10-year-olds; 45		12- vs. 13-year-olds: 0.44 (-0.01, 0.89)
	11-year-olds; 29		
	12-year-olds; 41		6- vs. 14-year-olds: 1.97 (1.07, 2.87)
			7- vs. 14-year-olds: 2.07 (1.29, 2.85)
	13-year-olds; 35		8- vs. 14-year-olds: 1.38 (0.65, 2.11)
	14-year-olds; 11		9- vs. 14-year-olds: 1.19 (0.43, 1.95)
			10- vs. 14-year-olds: 1.37 (0.66, 2.08)
			11- vs. 14-year-olds: 2.61 (1.69, 3.53)
			12- vs. 14-year-olds: 0.74 (0.05, 1.43)
Chivers et al. [43]	993; N/A; 10–14	sSSB: one-legged stance, both legs, eyes open, eyes closed; total time in balance (s)	10- vs. 14-year-olds: 0.63 (0.49, 0.77)
	10-year-olds: 507		
	14-year-olds: 486		
Condon & Cremin [31]	495; F (275), M (220); 6–15	sSSB: one-legged stance, dominant leg, firm surface, eyes open; time in balance (s)	6-7- vs. 12-15-year-olds: 2.33 (2.02, 2.64)
			8-9- vs. 12-15-year-olds: 1.37 (1.12, 1.62)
	6-7-year-olds; 125		10-11- vs. 12-15-year-olds: 0.36 (0.09, 0.63)
	8-9-year-olds; 152		
	10-11-year-olds; 78		
	12-15-year-olds; 140		
Donahoe et al. [36]	95; F (53), M (42); 7–15	PB: FR; mean reach (cm)	7-8- vs. 13-15-year-olds: 0.80 (0.07, 1.53)
			9-10- vs. 13-15-year-olds: 0.81 (-0.03, 1.65)
	7-8-year-olds; 36		11-12- vs. 13-15-year-olds: -0.06 (-0.77, 0.65)
	9-10-year-olds; 15		
	11-12-year-olds; 34		
	13-15-year-olds; Mid-PHV; 10		
Habib et al. [51]	160; F (80), M (80); 6–13	PB: FR; mean reach (cm)	6- vs. 13-year-olds: 1.01 (0.34, 1.68)
			7- vs. 13-year-olds: 1.14 (0.45, 1.83)
	6-year-olds; 20		8- vs. 13-year-olds: 0.53 (-0.12, 1,18)
	7-year-olds; 20		9- vs. 13-year-olds: 0.85 (0.18, 1.52)
	8-year-olds; 20		10- vs. 13-year-olds: 0.71 (0.06, 1.36)
	9-year-olds; 20		11- vs- 13-year-olds: 0.28 (-0.35, 0.91)
	10-year-olds; 20		12- vs- 13-year-olds: 0.24 (-0.39, 0.87)
	11-year-olds; 21		
	12-year-olds; 20		
	13-year-olds; 19		
Hirabayashi & Iwasaki [10]	79; F (39), M (40); 7–15	sSSB: SOT; total equilibrium score	7-8- vs. 14-15-year-olds: 1.71 (0.95, 2.47)
			9-10- vs. 14-15-year-olds: 1.37 (0.68, 2.06)
	7-8-year-olds; 18		11-13- vs. 14-15-year-olds: 0.30–0.33, 0.93)
	9-10-year-olds; 22		
	11-13-year-olds; 20		
	14-15-year-olds; Mid-PHV; 19		
Itzkowitz et al. [52]	1295; F (743), M (552); 6–13	PB: TUG; time (s)	6- vs.13-year-olds: -0.29 (-0.60, 0.02)
			7- vs.13-year-olds: -0.31 (-0.62, 0.00)
	6-year-olds; 244		8- vs.13-year-olds: -0.68 (-1.01, -0.35)
	7-year-olds; 221		9- vs.13-year-olds: -0.91 (-1.24, -0.58)
	8-year-olds; 197		10- vs.13-year-olds: -0.89 (-1.22, -0.56)
	9-year-olds; 203		11- vs.13-year-olds: -0.70 (-1.07, -0.33)
	10-year-olds; 180		12- vs.13-year-olds: -0.44 (-0.79, -0.09)
	11-year-olds; 95		
	12-year-olds; 110		
	13-year-olds; Mid-PHV; 45		
Johnson et al. [44]	33; N/A; 10.35 ± 3.28	sSSB: one-legged stance, dominant leg, eyes open; sway velocity in anterior/posterior directions (mm/s)	8-12- vs. 13-18-year-olds: 0.84 (0.04, 1.64)
	8-12-year-olds; 19		
	13-18-year-olds; 14		
Khanna et al. [45]	100; F (35), M (65); 12.47 ± 2.01	sSSB: BESS; total score	10-13- vs. 14-17-year-olds: 0.07 (-0.34, 0.48)
	10-13-years-olds; 68		
	14-17-years-olds; 32		
Menkveld et al. [50]	60; N/A; 7–16	dSSB: gait analysis using Electrodynogram and force data collector, walking at self-selected speed; cadence	7-8- vs. 13-14-year-olds: 2.25 (1.19, 3.31)
			9-10- vs. 13-14-year-olds: 0.91 (0.07, 1.75)
	7-8-year-olds; 12		11-12- vs. 13-14-year-olds: 0.50 (-0.32, 1.32)
	9-10-year-olds; 12		
	11-12-year-olds; 12		7-8- vs. 15-16-year-olds: 2.59 (1.45, 3.73)
			9-10- vs. 15-16-year-olds: 1.17 (0.29, 2.05)
	13-14-year-olds; 12		11-12- vs- 15-16-year-olds: 1.01 (0.15, 1.87)
	15-16-year-olds; 12		
Müller et al. [49]	5,215; F (2,771), M (2,444); 6–15	dSSB: gait analysis using VICON, force plates and photo electronic barrier; gait velocity (m/s)	6- vs. 13-year-olds: 0.32 (0.16, 0.48)
	6-year-olds; 977		7- vs. 13-year-olds: 0.28 (0.12, 0.44)
	7-year-olds; 834		8- vs. 13-year-olds: 0.16 (0.00, 0.32)
	8-year-olds; 819		9- vs. 13-year-olds: 0.24 (0.08, 0.40)
	9-year-olds; 711		10- vs. 13-year-olds: 0.15 (-0.01, 0.31)
	10-year-olds; 687		11- vs. 13- year-olds: 0.11 (-0.07, 0.29)
	11-year-olds; 437		12- vs. 13-year-olds: 0.09 (-0.09, 0.27)
	12-year-olds; 364		
			6- vs. 14-year-olds: 0.31 (0.11, 0.51)
	13-year-olds; 197		7- vs. 14-year-olds: 0.27 (0.07, 0.47)
	14-year-olds; 109		8- vs. 14-year-olds: 0.15 (-0.05, 0.35)
	15-year-olds; 80		9- vs. 14-year-olds: 0.23 (0.03, 0.43)
			10- vs. 14-year-olds: 0.14 (-0.06, 0.34)
			11- vs. 14- year-olds: 0.10 (-0.12, 0.32)
			12- vs. 14-year-olds: 0.08 (-0.14, 0.30)
			6- vs. 15-year-olds: 0.40 (0.16, 0.64)
			7- vs. 15-year-olds: 0.36 (0.12, 0.60)
			8- vs. 15-year-olds: 0.24 (0.00, 0.48)
			9- vs. 15-year-olds: 0.33 (0.09, 0.57)
			10- vs. 15-year-olds: 0.24 (0.00, 0.48)
			11- vs. 15- year-olds: 0.19 (-0.05, 0.43)
			12- vs. 15-year-olds: 0.18 (-0.06, 0.42)
Nicolini-Panisson & Donadio [53]	385; F (199), M (186); 6–18	PB: TUG; time (s)	6-9- vs. 14-18-year-olds: 0.82 (0.57, 1.07)
	6-9-year-olds; 130		10-13- vs. 14-18-year-olds: 0.71 (0.46, 0.96)
	10-13-year-olds; 129		
	14-18-year-olds; 126		
Sheldon [5]	39; F (22), M (17); 6–19	sSSB: 60-s two-legged stance, eyes closed; number of squares penetrated by tracing	6-9- vs. 15-19-year-olds: 2.38 (1.52, 3.24)
	6-9-year-olds; 14		
	15-19-year-olds; 25		
Steindl et al. [17]	100; F (50), M (50); 7–16	sSSB: SOT; total equilibrium score	7-8- vs. 13-14-year-olds: 1.68 (0.95, 2.41)
			9-10- vs. 13-14-year-olds 1.03 (0.36, 1.70)
	7-8-year-olds; 20		11-12- vs. 13-14-year-olds: -0.20 (-0.83, 0.43)
	9-10-year-olds; 20		
	11-12-year-olds; 20		7-8- vs. 15-16-year-olds: 2.69 (1.81, 3.57)
			9-10- vs. 15-16-year-olds: 1.84 (1.10, 2.58)
	13-14-year-olds; 20		11-12- vs. 15-16-year-olds: 0.55 (-0.08, 1.18)
	15-16-year-olds; 20		
Tringali et al. [46]	56; F (25), M (31); 6–15	sSSB: 4 x 12.8-s two-legged stance in Romberg position, eyes open on a force platform; sway velocity (mm/s)	6-7- vs. 12-13-year-olds: 3.22 (1.91, 4.53)
			8-9- vs. 12-13-year-olds: 0.36 (-0.52, 1.24)
	6-7-year-olds; 14		10-11- vs. 12-13-year-olds: 0.06 (-0.80, 0.92)
	8-9-year-olds; 11		
	10-11-year-olds; 12		6-7- vs. 14-15-year-olds: 6.02 (3.98, 8.06)
			8-9- vs. 14-15-year-olds: 2.87 (1.58, 4.16)
	12-13-year-olds; 9		10-11- vs. 14-15-year-olds: 1.61 (0.61, 2.61
	14-15-year-olds; 10		
Volkman et al. [54]	80; F (40), M(40); 7–16	PB: FR; mean reach (cm)	7-8- vs. 15-16-year-olds: 1.99 (1.32, 2.66)
			11-12- vs. 15-16-year-olds: 0.45 (-0.10, 1.00)
	7-8-year-olds; 29		
	11-12-year-olds; 26		
	15-16-year-olds; 25		
Walchli et al. [55]	77; F (38), M (39); 6–15	sSSB: 15-s Romberg stance, eyes open on a force platform; total sway path (cm)	sSSB
			6-7- vs. 14-15-year-olds: 1.25 (0.64, 1.86)
	6-7-year-olds: 25		11-12- vs. 14-15-year-olds: 0.37 (-0.18, 0.92)
	11-12-year-olds: 27		
		dSSB: 15-s two-legged stance, eyes open on a spinning top (Pedalo Kreisel) placed on force platform; total sway path (cm)	dSSB
	14-15-year-olds: 25		6-7- vs. 14-15-year-olds: 1.18 (0.57, 1.79)
			11-12- vs. 14-15-year-olds: 0.36 (-0.19, 0.91)
		RB: 15-s two-legged stance, eyes open on swinging platform (Posturomed); total sway path (cm) assessed using VICON	RB
			6-7- vs. 14-15-year-olds: 1.12 (0.52, 1.71)
			11-12- vs. 14-15-year-olds: 0.10 (-0.45, 0.64)
Wolff et al. [47]	74; N/A; 7–18	sSSB: 10 x 30-s two-legged stance, barefoot, eyes open; sway velocity (mm/s)	7-8- vs. 13-14-year-olds: 0.64 (-0.12, 1.40)
			9-10- vs. 13-14-year-olds: 0.56 (-0.20, 1.32
	7-8-year-olds; 16		11-12- vs. 13-14-year-olds: 0.11 (-0.65, 0.87)
	9-10-year-olds; 16		
	11-12-year-olds; 15		7-8- vs. 15-18-year-olds: 1.05 (0.29, 1.81)
			9-10- vs. 15-18-year-olds: 0.91 (0.17, 1.65)
	13-14-year-olds; 12		11-12- vs. 15-18-year-olds: 0.38 (-0.35, 1.11)
	15-18-year-olds; 15		
Zaino et al. [37]	27; F (14), M (13); 8–14	PB: Timed up and down stairs test; time (s)	8-10- vs. 13-14-year-olds: 1.09 (0.11, 2.07)
			11-12- vs. 13-14-year-olds: 0.24 (-0.86, 1.34)
	8-10-year-olds; 14		
	11-12-year-olds; 6		
	13-14-year-olds; 7		
Zumbrunn et al. [48]	32; F (17), M (15); 8–18	sSSB: 5 x 5-s one-legged stance, dominant and non-dominant leg, eyes open; sway velocity (mm/s)	8-12- vs. 13-18-year-olds: 1.07 (0.31, 1.83)
	8-12-year-olds; 19		
	13-18-year-olds; 13		

*BESS* Balance error scoring system, *dSSB* dynamic steady-state balance, *F* female, *FR* Functional-Reach Test, *M* male, *N/A* not available, *PB* proactive balance, *SD* standard deviation, *SMD*_*ba*_ between-subject standardized mean difference (i.e., children versus adolescents), *SOT* Sensory Organization Test, *sSSB* static steady-state balance, *TUG* Timed up and go test.

Characteristics of the 25 included studies comparing measures of balance performance in boys and girls are shown in Table 4. Twelve studies [22, 24, 45, 56–64] examined variables of static steady-state balance, five studies [49, 65–68] analyzed outcomes of dynamic steady-state balance, four studies [23, 52, 69, 70] examined outcomes of proactive balance, two studies [21, 71] reported proxies of both static and dynamic steady-state balance, and two studies [72, 73] scrutinized measures of static steady-state and proactive balance. No study reported outcomes of reactive balance. Eligible for analysis were the data of a total of 10,723 subjects aged between six and 19 years of whom 5,143 were males and 5,580 were females.

**Table 4:** Included studies examining sex differences (boys vs. girls) in balance performance in youth.

References	No. of subjects; sex; age [years (range or mean ± SD)]; maturation; n	Balance test parameter; outcome	Results SMD_bs_ (95% CI)
Arévalo-Mora et al. [56]	187; F (97), M (90); 11.15 ± 1.24	sSSB: 60-s one-legged stance, barefoot, eyes open on beam (3 cm wide, 20 cm above floor); time in balance (s)	0.09 (-0.20, 0.38)
Butz et al. [23]	140; F (70), M (70); 6–12	PB: TUG; time (s)	6-year-olds: 0.57 (-0.33, 1.47)
			7-year-olds: 0.26 (-0.62, 1.14)
	6-year-olds; F (10), M (10)		8-year-olds: -0.51 (-1.41, 0.39)
	7-year-olds; F (10), M (10)		9-year-olds: 0.50 (-0.40, 1.40)
	8-year-olds; F (10), M (10)		10-year-olds: -0.19 (-1.07, 0.69)
	9-year-olds; F (10), M (10)		11-year-olds: -0.51 (-1.41, 0.39)
	10-year-olds; F (10), M (10)		12-year-olds: 0.51 (-0.39, 1.41)
	11-year-olds; F (10), M (10)		
	12-year-olds; F (10), M (10)		
Davies & Rose [57]	60; F (30), M (30); 7–18	sSSB: one-legged stance, dominant leg, eyes open, firm surface; time in balance (20 s maximum)	Pre-PHV: -0.42 (-1.30, 0.46)
			Mid-PHV: -0.42 (-1.30, 0.46)
	Females		Post-PHV: 0.42 (-0.46, 1.30)
	Pre-PHV; 9.55 ± 0.72; 10		
	Mid-PHV; 12.65 ± 1.36; 10		
	Post-PHV; 16.14 ± 1.09; 10		
	Males		
	Pre-PHV; 11.28 ± 1.26; 10		
	Mid-PHV; 14.51 ± 1.32; 10		
	Post-PHV; 17.74 ± 0.85; 10		
Deshmukh et al. [69]	350; F (175), M (175); 6–12	PB: FR; mean reach (cm)	6-year-olds: -0.67 (-1.24, -0.10)
			7-year-olds: -0.76 (-1.33, -0.19)
	6-year-olds; F (25), M (25)		8-year-olds: 0.06 (-0.49, 0.61)
	7-year-olds; F (25), M (25)		9-year-olds: -0.39 (-0.96, 0.18)
	8-year-olds; F (25), M (25)		10-year-olds: -0.16 (-0.71, 0.39)
	9-year-olds; F (25), M (25)		11-year-olds: -0.14 (-0.69, 0.41)
	10-year-olds; F (25), M (25)		12-year-olds: 0.68 (0.11, 1.25)
	11-year-olds; F (25), M (25)		
	12-year-olds; F (25), M (25)		
Dufek et al. [65]	56; F (23), M (33); 14.7 ± 1.5	dSSB: gait analysis using GAITRite system; gait velocity during preferred walking speed (m/s)	0.17 (-0.36, 0.70)
Eguchi & Takada [21]	47; F (23), M (24); 8–11	sSSB: 2 x 30-s one-legged stance, barefoot, dominant leg; mean synthesized	sSSB
		root mean square (S-RMS) of body accelerations	9-year-olds: 1.08 (0.18, 1.98)
	sSSB		11-year-olds: 0.80 (-0.10, 1.70)
	9-year-olds; F (11), M (11)		
	11-year-olds; F (10), M (11)	dSSB: 2 x 5 m walking, barefoot self-selected speed; mean synthesized root mean square (S-RMS) of body accelerations	dSSB
			9-year-olds: 0.48 (-0.32, 1.28)
	dSSB		11-year-olds: 0.33 (-0.51, 1.17)
	9-year-olds; F (12), M (13)		
	11-year-olds: F (11), M (11)		
Geldhof et al. [71]	99; F (58), M (41); 9.8 ± 0.5	sSSB: modified clinical test of sensory interaction on balance; sway velocity (°/sec)	sSSB
			0.59 (0.18, 1.00)
		dSSB: LOS; sway velocity (°/sec)	dSSB
			-0.04 (-0.43, 0.35)
Holm & Vøllestad [58]	368; F (184), M (184); 7–12	sSSB: one-legged stance on KAT 2000 system; Balance Index	7-year-olds: 0.70 (0.13, 1.27)
			8-year-olds: 0.07 (-0.46, 0.60)
	7-year-olds; F(19), M (40)		9-year-olds: 0.43 (-0.06, 0.92)
	8-year-olds; F (26), M (29)		10-year-olds: 0.06 (-0.41, 0.53)
	9-year-olds; F (39), M (29)		11-year-olds: 0.82 (0.29, 1.35)
	10-year-olds; F (40), M (29)		12-year-olds: 0.37 (-0.14, 0.88)
	11-year-olds; F (29), M (29)		
	12-year-olds; F (31), M (28)		
Holm et al. [66]	360; F (179), M (181); 7–12	dSSB: gait analysis using GAITRite; cadence (step/min) at normalized gait velocity of 1.5 m/s	7-year-olds: 0.00 (-0.59, 0.59)
			8-year-olds: -0.46 (-1.03, 0.11)
	7-year-olds: 52; F (16), M (36)		9-year-olds: 0.23 (-0.24, 0.70)
	8-year-olds: 48; F (21), M (27)		10-year-olds: -0.10 (-0.59, 0.39)
	9-year-olds: 69; F (38), M (31)		11-year-olds: -0.40 (-0.95, 0.15)
	10-year-olds: 68; F (39), M (29)		12-year-olds: 0.00 (-0.47, 0.47)
	11-year-olds: 53; F (28), M (25)		
	12-year-olds: 70; F (37), M (33)		
Itzkowitz et al. [52]	1295; F (743), M (552); 6–13	PB: TUG; time (s)	6-year-olds: -0.06 (-0.31, 0.19)
			7-year-olds: -0.03 (-0.30, 0.24)
	6-year-olds; F (120), M (124)		13-year-olds: 0.14 (-0.57, 0.85)
	7-year-olds; F (129), M (92)		8-year-olds: -0.44 (-0.71, -0.17)
	8-year-olds; F (98), M (99)		9-year-olds: -0.58 (-0.87, -0.29)
	9-year-olds; F (130), M (73)		10-year-olds: -0.26 (-0.55, 0.03)
	10-year-olds; F (112), M (68)		11-year-olds: -0.50 (-0.91, -0.09)
	11-year-olds; F (45), M (50)		12-year-olds: -0.05 (-0.44, 0.34)
	12-year-olds; F (74), M (36)		
	13-year-olds; F (35), M (10)		
Khanna et al. [45]	100; F (35), M (65); 12.47 ± 2.01	sSSB: BESS; total score	0.19 (-0.22, 0.60)
Laguna Nieto et al. [73]	18; F (9), M (9); N/A	sSSB: 10-s one-legged stance on a force platform, right leg, barefoot, eyes open; total sway area (mm^2^)	sSSB
			1.77 SE 0.58 (0.63, 2.91)
		PB: moving CoP towards specified targets as fast as possible; accuracy (%)	PB
			0.41 SE 0.48 (-0.53, 1.35)
Lee & Lin [59]	709; F (344), M (365); 9.61 ± 0.68	sSSB: 3 x 10-s one-legged stance, barefoot, alternately on left and right leg, eyes open on a force platform; mean radius of CoP (cm)	0.25 (0.19, 0.41)
Libardoni et al. [24]	165; F (82), M (83); 8–12	sSSB: SOT, balance score in condition one (two-legged stance, eyes open)	8-year-olds: -0.51 (-1.49, 0.47)
			9-year-olds: 0.18 (-0.58, 0.94)
			10-year-olds: 0.69 (-0.21, 1.59)
			11-year-olds: -0.14 (-1.16, 0.88)
			12-year-olds: -0.53 (-1.43, 0.37)
McKay et al. [72]	160; F (80), M (80); 10–19	sSSB: one-legged stance, eyes closed; time in balance (maximum 10 s)	sSSB: 0.21 (-0.10, 0.52)
		PB: star excursion balance test; distance reached in posteromedial direction (% of leg length)	PB: -0.24 (-0.55, 0.07)
Michelotti et al. [60]	52; F (24), M (28); 13.73 ± 1.20 (mean age of	sSSB: 51.2-s two-legged stance on a force platform; sway velocity (mm/s)	0.37 (-0.18, 0.92)
	matched intervention group)		
Mickle et al. [61]	84; F (47), M (37); 8–12	sSSB: 30-s one-legged stance, dominant leg, eyes open; body sway assessed via Lord sway-meter	0.53 (0.10, 0.96)
Müller et al. [49]	5,215; F (2,771), M (2,444); 6–15	dSSB: gait analysis using VICON, force plates and photo electronic barrier; gait velocity (m/s)	6-year-olds: 0.11 (-0.01, 0.23)
			7-year-olds: 0.04 (-0.10, 0.18)
	6-year-olds; F (522), M (455)		8-year-olds: -0.08 (-0.22, 0.06)
	7-year-olds; F (426), M (408)		9-year-olds: -0.13 (-0.29, 0.03)
	8-year-olds; F (434), M (385)		10-year-olds: -0.10 (-0.26, 0.06)
	9-year-olds; F (391), M (320)		11-year-olds: -0.06 (-0.26, 0.14)
	10-year-olds; F (372), M (315)		12-year-olds: -0.09 (CI -0.29, 0.11)
	11-year-olds; F (237), M (200)		13-year-olds: -0.01 (-0.28, 0.26)
	12-year-olds; F (184), M (180)		14-year-olds: -0.19 (-0.56, 0.18)
	13-year-olds; F (99), M (98)		15-year-olds: 0.13 (-0.32, 0.58)
	14-year-olds; F (57), M (52)		
	15-year-olds; F (49), M (31)		
Öberg et al. [67]	54; F (27), M (27); 10–19	dSSB: gait analysis, walking at normal speed; gait velocity (cm/s)	10-14-year-olds: -1.43 (-2.35, -
			0.51)
	10-14-year-olds: F (12), M (12)		15-19-year-olds: -0.70 (-1.44, 0.04)
	15-19-year-olds: F (15), M (15)		
Peterson et al. [62]	154; F (74), M (80); 6–12	sSSB: SOT; total equilibrium score	6-year-olds: 0.97 (-0.48, 2.42)
			7-year-olds: 0.60 (-0.18, 1.38)
	6-year-olds; F (4), M (5)		8-year-olds: 0.25 (-0.44, 0.94)
	7-year-olds; F (14), M (12)		9-year-olds: 0.38 (-0.29, 1.05)
	8-year-olds; F (14), M (21)		10-year-olds: -0.20 (-1.08, 0.68)
	9-year-olds; F (20), M (16)		11-year-olds: 0.76 (-0.22, 1.74)
	10-year-olds; F (9), M (11)		12-year-olds: 0.02 (-1.29, 1.33)
	11-year-olds; F (7), M (11)		
	12-year-olds; F (5), M (4)		
Sheehan & Katz [64]	65; F (36), M (29); 6–9	sSSB: 6 x 20-s stance (one-legged stance, eyes open, firm surface; one-legged stance eyes closed, firm surface; one-legged stance, eyes open, foam surface; tandem stance, eyes open, firm surface; tandem stance, eyes closed, firm surface; tandem stance, eyes open, foam surface); total CoP path (mm)	0.61 (0.10, 1.12)
Sheehan & Katz [63]	61; F (28), M (33); N/A	sSSB: 6 x 20-s stance (one-legged stance, eyes open, firm surface; one-legged stance eyes closed, firm surface; one-legged stance, eyes open, foam surface; tandem stance, eyes open, firm surface; tandem stance, eyes closed, firm surface; tandem stance, eyes open, foam surface); total CoP path (mm)	0.49 (-0.02, 1.00)
Smith et al. [22]	26; F (9), M (17); 8–12	sSSB: 3 x 30-s two-legged stance, barefoot; sway velocity (mm/s)	0.55 (-0.27, 1.37)
Thevenon et al. [68]	382; F (154), M (228); 6–12	dSSB: gait analysis using GAITRite; gait velocity (cm/s)	6-year-olds: -0.12 (-0.65, 0.41)
			7-year-olds: 0.32 (-0.25, 0.89)
	6-year-olds: 61; F (20), M (41)		8-year-olds: -0.35 (-0.90, 0.20)
	7-year-olds: 53; F (20), M (33)		9-year-olds: 0.22 (-0.31, 0.75)
	8-year-olds: 56; F (22), M (34)		10-year-olds: 0.33 (-0.22, 0.88)
	9-year-olds: 57; F (27), M (30)		11-year-olds: 0.43 (-0.14, 1.00)
	10-year-olds: 54; F (20), M (34)		12-year-olds: 0.87 (0.26, 1.48)
	11-year-olds: 54; F (20), M (34)		
	12-year-olds: 47; F (25), M (22)		
Yuksel et al. [70]	280; F (152), M (128); 6–12	PB: FR; mean reach (cm)	6-year-olds: 0.48 (-0.15, 1.11)
			7-year-olds: 0.42 (-0.23, 1.07)
	6-year-olds; F (20), M (20)		8-year-olds: -0.51 (-1.14, 0.12)
	7-year-olds; F (25), M (15)		9-year-olds: 0.00 (-0.63, 0.63)
	8-year-olds; F (20), M (20)		10-year-olds: -0.21 (-0.84, 0.42)
	9-year-olds; F (22), M (18)		11-year-olds: -0.15 (-0.78, 0.48)
	10-year-olds; F (21), M (19)		12-year-olds: -0.40 (-1.03, 0.23)
	11-year-olds; F (23), M (17)		
	12-year-olds; F (21), M (19)		

*BESS* Balance error scoring system, *CoP* center of pressure, *dSSB* dynamic steady-state balance, *F* female, *FR* Functional-Reach Test, *LOS* limits of stability, *M* male, *N/A* not available, *PB* proactive balance, *PHV* peak height velocity, *SD* standard deviation, *SMDbs* between-subject standardized mean difference (i.e., boys versus girls), *SOT* Sensory Organization Test, *sSSB* static steady-state balance, *TUG* Timed up and go test.

### Quality of the included studies

Quality assessment revealed that the majority of included studies complied with most of the criteria of the Appraisal tool for Cross-Sectional studies. In detail, 40 out of 43 studies fulfilled ≥4 out of 7 criteria regarding the quality of study reports, 43 out of 43 studies fulfilled ≥4 out of 7 criteria addressing the design of studies, and concerning risk of bias ≥2 out of 3 criteria were met by 40 out of the 43 included studies (S1 Table). Thus, the majority of studies met the criteria above average.

### Age differences

**Fig 2:**
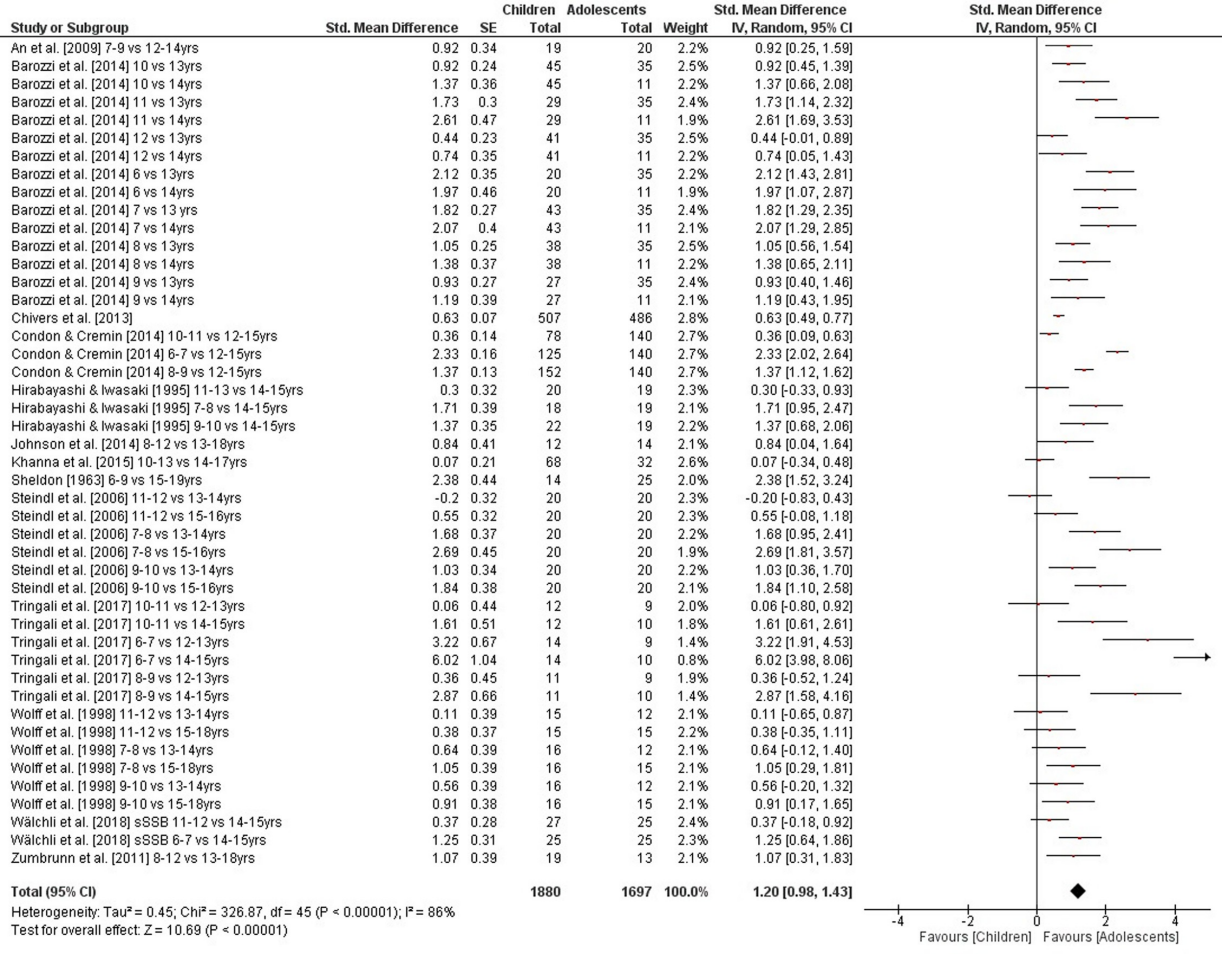
Differences in measures of static steady-state balance by age (children vs. adolescents). *CI* confidence interval, *df* degrees of freedom, *SE* standard error, *IV* inverse variance.

**Fig 3:**
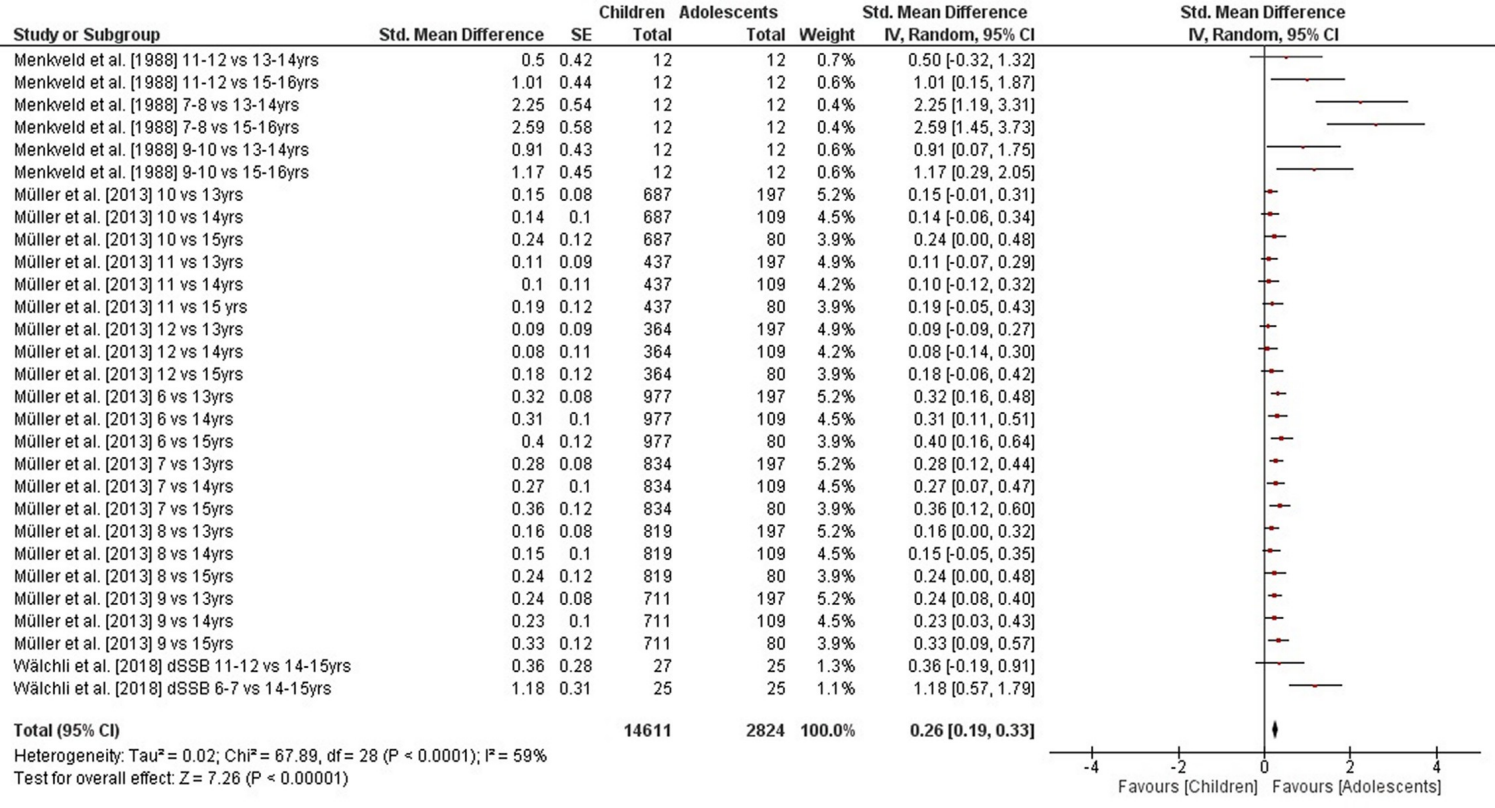
Differences in measures of dynamic steady-state balance by age (children vs. adolescents). *CI* confidence interval, *df* degrees of freedom, *SE* standard error, *IV* inverse variance.

**Fig 4:**
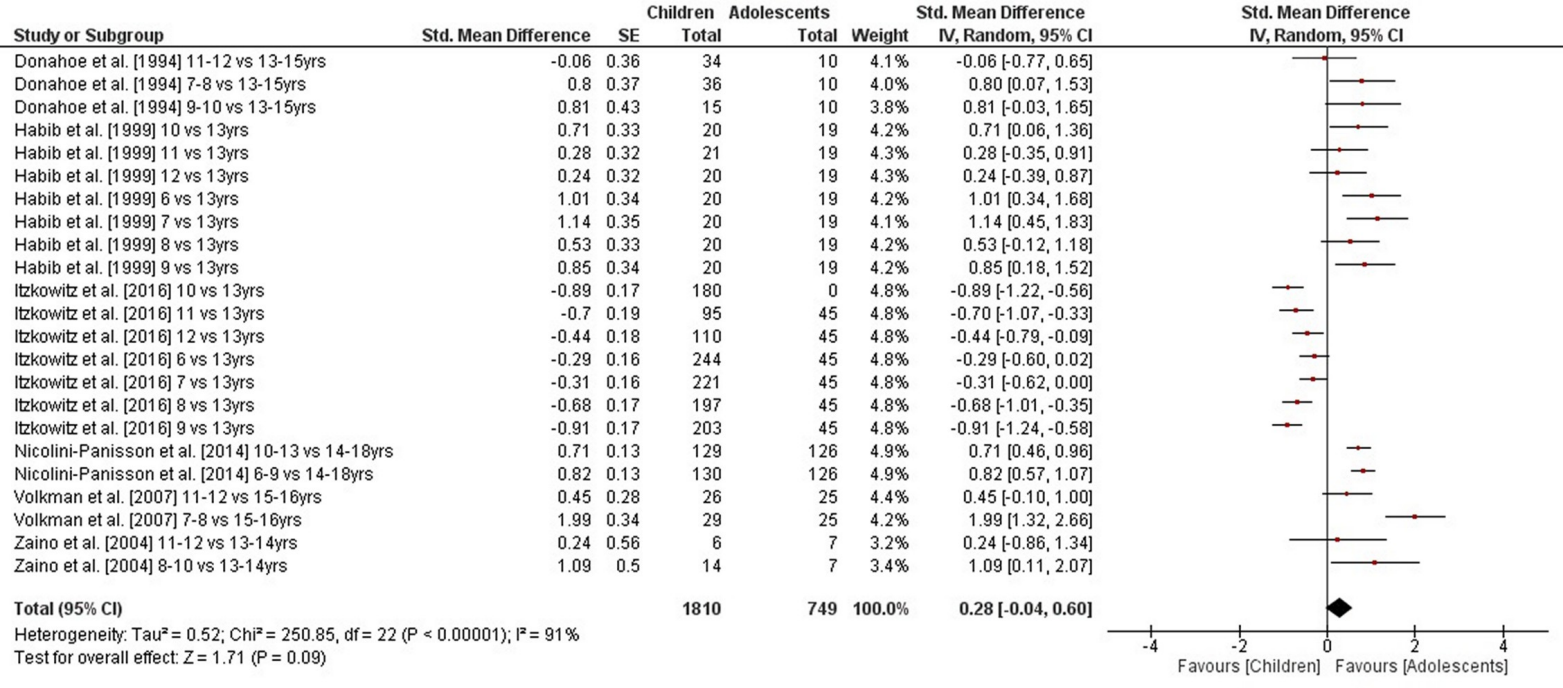
Differences in measures of proactive balance by age (children vs. adolescents). *CI* confidence interval, *df* degrees of freedom, *SE* standard error, *IV* inverse variance.

The comparisons of static steady-state balance performance between children and adolescents is shown in Fig 2. Weighted mean SMD_ba_ amounted to 1.20 (*I*^2^ = 86%, Chi^2^ = 326.87, *df* = 45, *p* < .001, 13 studies, 46 comparisons) suggesting a large effect in favor of adolescents. Moreover, weighted mean SMD_ba_ amounted to 0.26 for outcomes of dynamic steady-state balance (*I*^2^ = 59%, Chi^2^ = 67.89, *df* = 28, *p* < .001, three studies, 29 comparisons) and to 0.28 for variables of proactive balance (*I*^2^ = 91%, Chi^2^ = 250.85, *df* = 22, *p* < .001, six studies, 23 comparisons), respectively, indicating small-sized effects in favor of adolescents (Figs 3 and 4). Heterogeneity between studies was considerable for static steady-state and proactive balance and substantial in terms of dynamic steady-state balance. No SMD_ba_ was calculated for reactive balance as our search did not identify the required minimum of two studies.

### Sex differences

**Fig 5:**
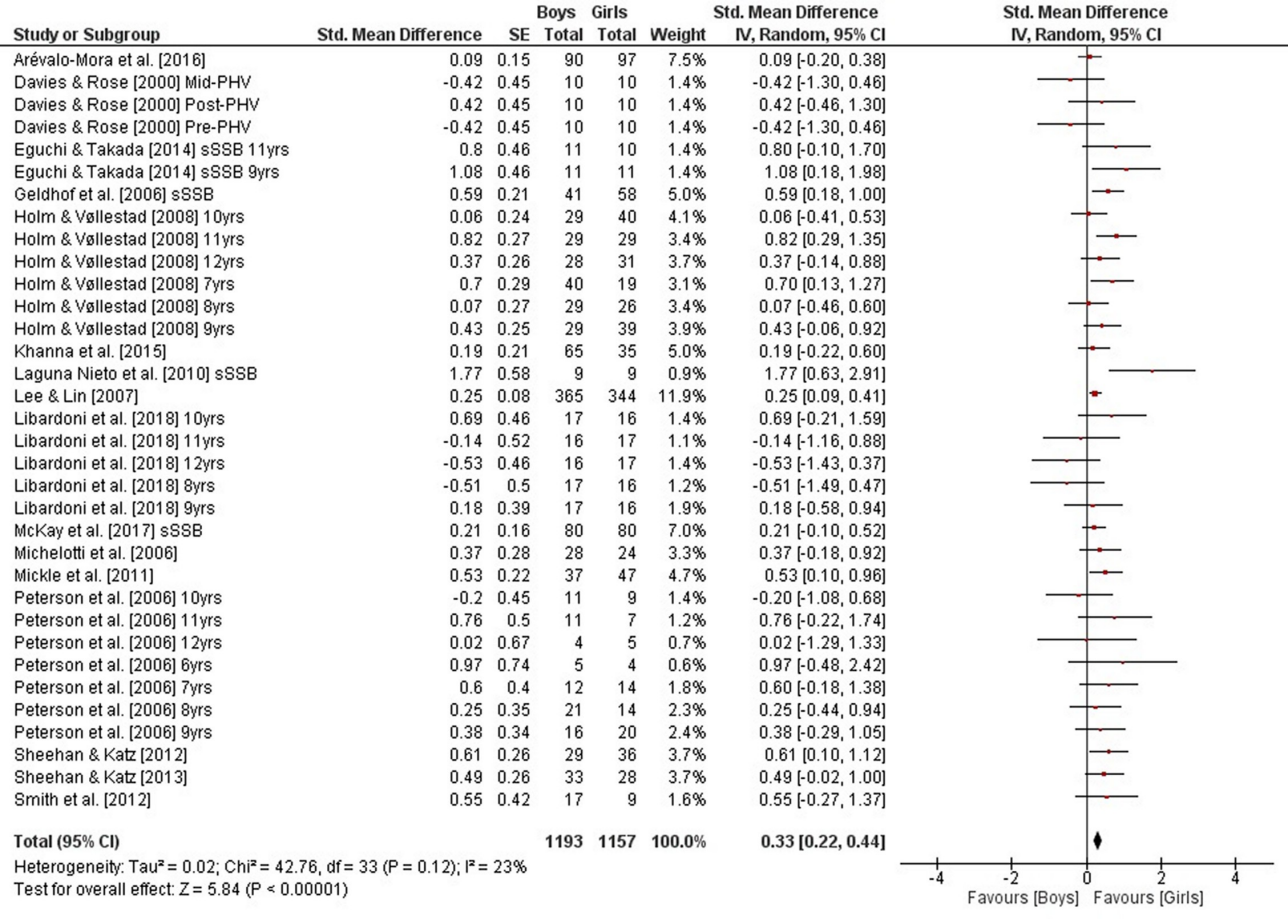
Differences in measures of static steady-state balance by sex (boys vs. girls). *CI* confidence interval, *df* degrees of freedom, *SE* standard error, *IV* inverse variance.

**Fig 6:**
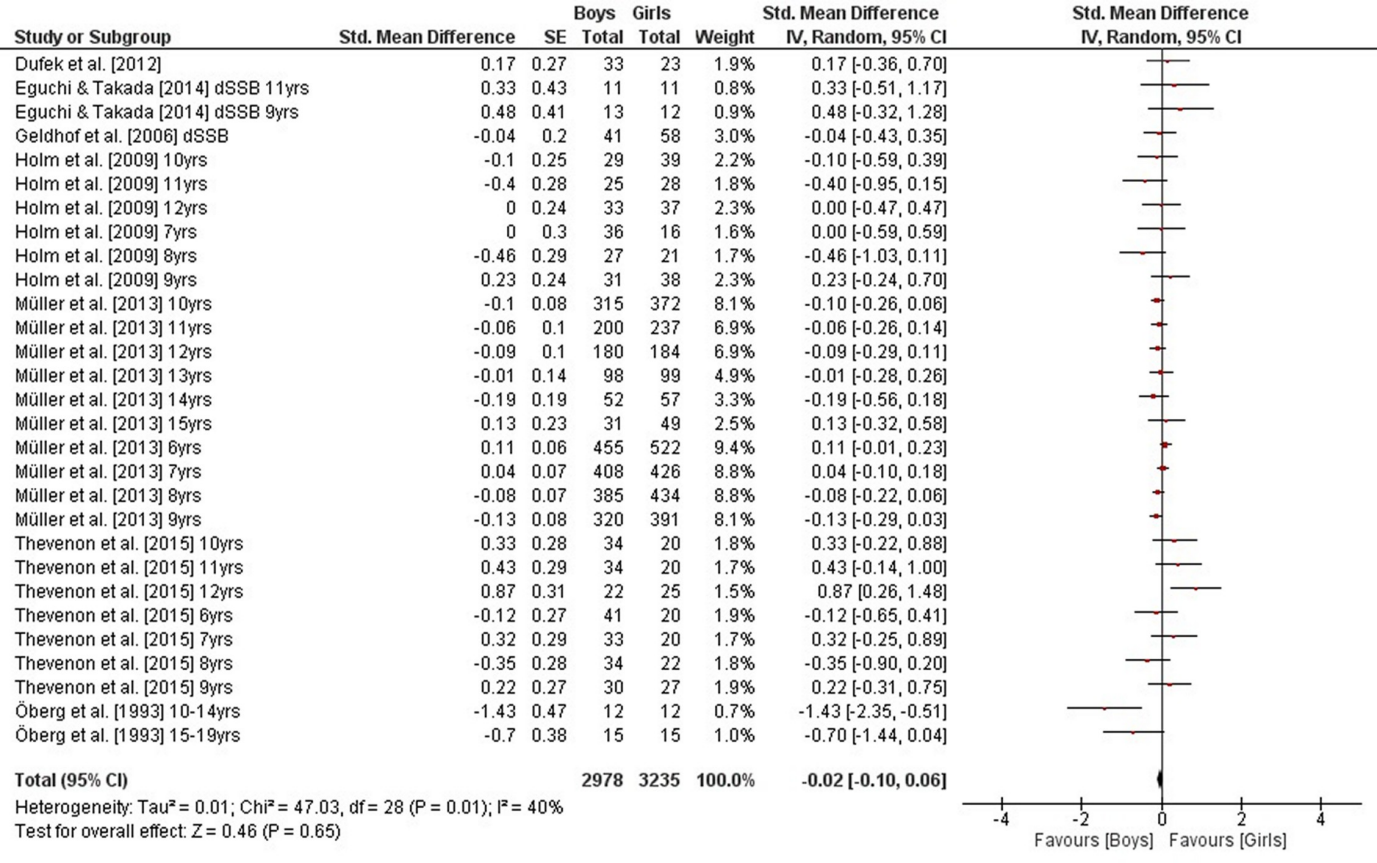
Differences in measures of dynamic steady-state balance by sex (boys vs. girls). *CI* confidence interval, *df* degrees of freedom, *SE* standard error, *IV* inverse variance.

**Fig 7:**
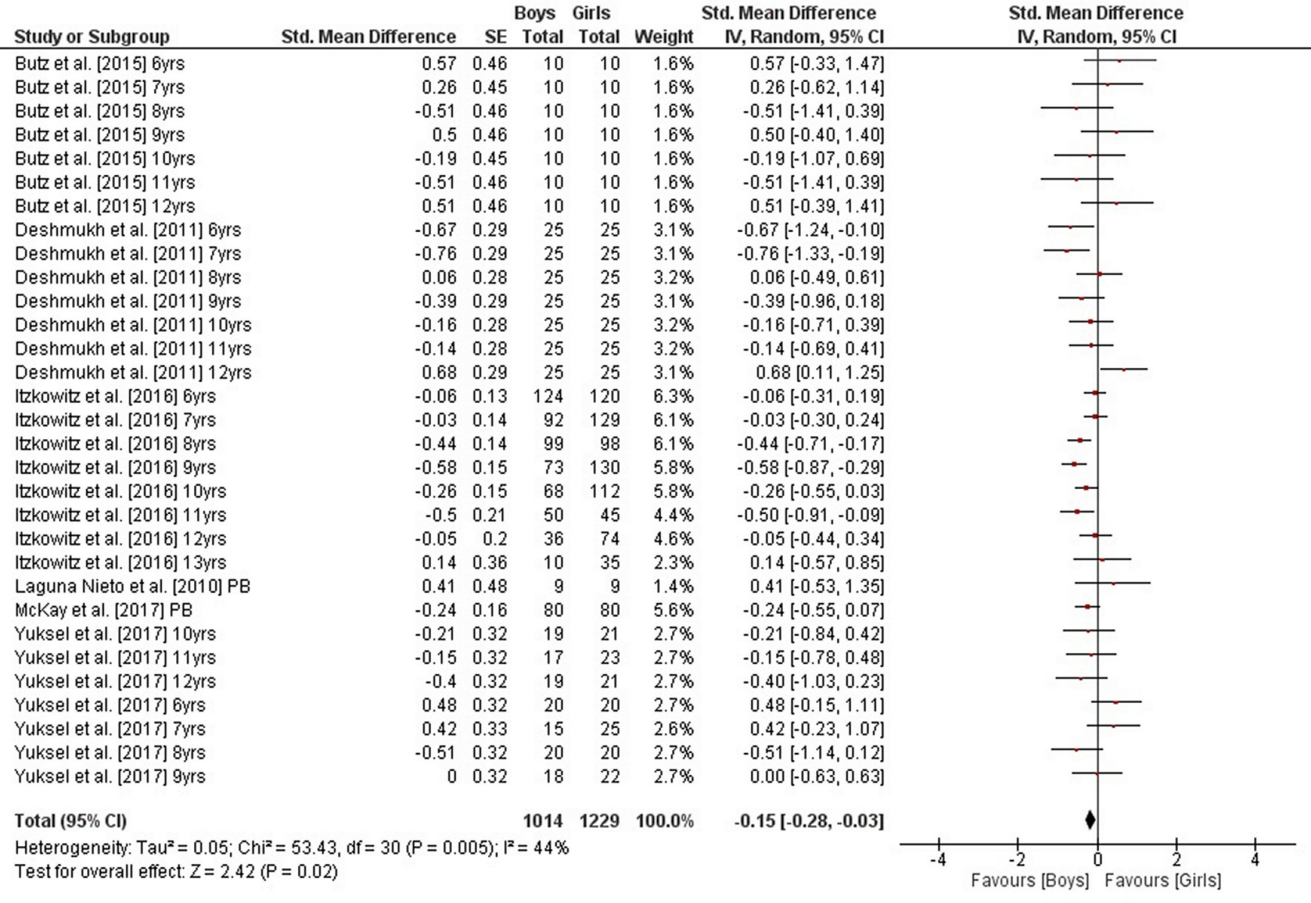
Differences in measures of proactive balance by sex (boys vs. girls). *CI* confidence interval, *df* degrees of freedom, *SE* standard error, *IV* inverse variance.

As illustrated in Fig 5, weighted mean SMD_bs_ amounted to 0.33 (*I*^2^ = 23%, Chi^2^ = 42.76, *df* = 33, *p* = 0.12, sixteen studies, 34 comparisons) for measures of static steady-state balance, indicating a small effect in favor of girls. In contrast and as shown in Figs 6 and 7, small-sized effects in favor of boys were found for variables of dynamic steady-state and proactive balance as SMD_bs_ amounted to -0.02 (*I*^2^ = 40%, Chi^2^ = 47.03, *df* = 28, *p* = 0.01, seven studies, 29 comparisons) and -0.15 (*I*^2^ = 44%, Chi^2^ = 53.43, *df* = 30, *p* = 0.005, six studies, 31 comparisons), respectively. Heterogeneity between studies was trivial regarding static steady-state balance and moderate in terms of dynamic steady-state and proactive balance. Given that our literature search did not identify the required minimum of two studies on reactive balance no SMD_bs_ was calculated for that particular parameter. Changes in SMD_bs_ when calculated for children and adolescents separately were neglectable (data not shown).

## Discussion

To the best of our knowledge, the present systematic review and meta-analysis is the first which characterized and quantified age and sex differences in balance performance in youth. Analyses of the data of 43 studies revealed consistently better balance performances in adolescents compared to children irrespective of the variable considered, supporting our first hypothesis. Largest differences were found for measures of static steady-state balance, while SMD_ba_ were considerably smaller for measures of dynamic steady-state and proactive balance. In terms of sex differences in balance performance, SMD_bs_ revealed only small-sized effects. Girls showed better performances than boys in measures of static steady-state balance but were outperformed by boys in proactive balancing tasks. Even though boys showed slightly better performances than girls in terms of dynamic steady-state balance, this difference probably is functionally irrelevant due to the low SMD_bs_ of -0.02. Based on these results, we can only partially confirm our second hypothesis that girls show better balance performances compared to same-aged boys.

### Age differences in balance performance in youth

Our analysis revealed better balance performances of adolescents compared to children in terms of static steady-state, dynamic steady-state, and proactive balance. These findings support our first hypothesis that adolescents exhibit better postural control than children and disagree with studies indicating balance performance to be mature at around ten years of age [1, 74]. We conclude that maturation of balance may not be completed in childhood but possibly continue throughout adolescence in healthy youth.

The age-period between six and eight years of age is considered a transition phase in the development of postural control [15, 75]. At this stage, balance performance sharply increases which has been attributed to better sensory integration and reweighting [76, 77] as well as changes in postural control strategies [11, 12]. Compared to younger peers, it has been shown that children aged six and older perform distinctively better in situations where the equilibrium is perturbed or sensory input is suppressed and conflicting. Furthermore, postural control is gradually organized in a feedforward, anticipatory way and not solely controlled by feedback [78]. Several authors [9, 15, 79] found children at the end of the first decade of life to perform equally well as adults during balancing tasks. They concluded that postural control is mature at this age, although significant developments take place during adolescence also influencing balance performance. Correspondingly, other studies [8, 10, 62] reported further improvements of balance performance throughout adolescence which is in line with our findings.

Increased muscle strength and better attentional capabilities (i.e., to increase focus on a given balance task or challenging situation) may contribute to improved balance performances of adolescents compared to children. For instance, functional measurement tools such as the functional reach test are associated with lower extremity muscular strength in youth [80], potentially giving a slight advantage to older subjects. Moreover, most balance tasks demand high attention of the subject which might be difficult for (younger) children. Walchli et al. [55] for instance limited the test duration of balancing tasks in their study to 15 s in order to minimize bias due to decreased attention of children.

With regard to the results of our meta-analysis, one could conclude that differences in balance performance between children and adolescents might also lead to age-related differences in the trainability of balance in youth. This could be either in favour of children due to highe adaptive reserves or in favour of adolescsents due to their more mature postural control system. There is preliminary evidence that age influences adaptations to anticipated and non-anticipated perturbations in youth after balance training. Walchli et al. [81] compared adaptations to anticipated and non-anticipated perturbations between six- and twelve-year-olds after both age groups received five weeks of child oriented balance training and found younger children to improve similarly in anticipated and non-anticipated perturbations whereas the older children tended to show greater improvements in anticipated perturbations.

Further research investigating the effect of age on balance and its trainability in youth is needed to attain deeper knowledge of the processes underlying the development of postural control and to assist practitioners and clinicians with effective, age-based training regimes. As physical and neural maturation can markedly differ inter-individually, there is a need for large cohort, longitudinal studies on the development of balance performance in youth ideally using MRI as well as balance tests.

### Sex differences in balance performance in youth

Analysis of balance performance in youth with regard to a possible gender effect yielded inconsistent results. Being a girl was favorable for measures of static steady-state balance, whereas boys performed better in terms of proactive balance. With respect to dynamic steady-state balance differences between girls and boys seem negligible. Therefore, our second hypothesis that girls exhibit better balance performance than boys is only partially supported by the results.

Maturational differences between girls and boys do not only exist regarding physical, hormonal, and sexual development, but also in terms of central nervous structures [26]. Maturation of the CNS is, amongst others, characterized by a decrease in grey matter volume [14] and increases in white matter volume [14] as well as total cerebellar volume [82]. Similar to Marshalls’ and Tanners’ finding that height velocity on average peaks two years earlier in girls than in boys [83, 84], brain developmental studies have shown that brain maturation (e.g., gray matter decrease) occurs in girls prior to boys and is probably also affected by hormonal changes (e. g., sex steroids) accompanying puberty [85]. Consequently, girls reach their peak brain volume earlier than boys [86]. However, these findings must be interpreted carefully as for example regional volumes have to be adjusted for total brain size, which is usually smaller in girls, although not equivalent to any functional differences [13]. Furthermore, earlier brain maturation in girls might give a general advantage to them over boys but not necessarily in terms of balance as many of the described structures are not primarily involved in balance. Lastly, it is difficult to discriminate between effects of development and general sex-effects [86].

From a developmental perspective, it seems reasonable to argue that sex-related differences in balance performance in youth may also be age-dependent. At the beginning of the second decade of life, girls might exhibit better balance performance as they have usually already entered puberty which also leads to advanced maturation of the CNS, while boys typically lag behind in terms of physical and neural development. Contrary, in adolescents aged 15 years for example sex-related differences in balance performance might be smaller as disparities in the level of development are declining or have already been evened out. Therefore, we calculated separate SMD_bs_ for children and adolescents and assumed to find better balance performances of girls in children, but not necessarily in adolescents. However, neither comparison of sex-related balance performance with respect to age groups nor analyses of single SMD_bs_ by age revealed ages or age periods where girls particularly outperform boys or vice versa.

Sex-related differences in balance performance in youth were greatest for measures of static steady-state balance in favor of girls and for measures of proactive balance in favor of boys. Static steady-state balance (e.g., standing on one leg on firm surface) mainly requires the subject to use sensory information and stay concentrated in order to keep the center of mass steadily over the base of support. Contrary, proactive balance as for instance measured with FR requires the subject to move its center of mass to the limits of stability which may also involve other motor skills (e.g., muscular strength). Boys exhibit greater muscular strength than girls in childhood and adolescence and this difference increases with age [26]. Thus, boys may be at an advantage over girls in terms of proactive balance as they can compensate worse balance by greater muscle strength.

Altogether, the inconsistency of our results highlights the need for top-quality studies investigating sex-related differences in balance performance and balance training in youth.

### Limitations

A limitation of the present systematic review and meta-analysis is that the analysis of differences in balance performance was limited to the factors age and sex. Maturational processes also affecting the development of balance performance in youth differ in onset and velocity inter-individually, consequently complicating comparisons based on chronological age only. However, only one study [57] in the present meta-analysis reported biological age with subjects being either at the stage of pre-, mid-, or post-peak-height-velocity, leaving us unable to compare balance performances between children and adolescents and/or between girls and boys with respect to biological age, which limits the validity of our analysis. Therefore, future studies on balance performance in youth are advised to also assess biological age of their subjects.

Further, it cannot be ruled out that differences in physical activity between subjects might have influenced our results, although, we corrected for it by excluding data of athletes from our analyses. Thus, further research is needed to investigate differences in balance performance by additional factors like growth, maturation, physical activity, and expertise level.

Only one study [55] reported age- and no study reported sex-specific data for measures of reactive balance performance in youth, while the majority of included studies reported measures of static balance, which may be least important in youth from a functional perspective as daily life activities of children and adolescents (e.g., running, jumping) particularly involve dynamic abilities. Thus, future studies should focus more on dynamic, proactive, and reactive balance performance in boys and girls during childhood and adolescence.

Overall, we observed substantial to considerable heterogeneity between studies investigating age differences in balance performance in youth (*I*^*2*^ = 59–91%) and trivial to moderate in those on sex differences in balance performance in youth (*I*^*2*^ = 23–44%), which highlights the need for studies scrutinizing the development of balance performance in youth using the same experimental design in large cohorts covering broad numeric and biological age-ranges.

## Conclusions

The present systematic review and meta-analysis characterized and quantified age and sex differences in balance performance in youth. We found better performances in adolescents compared to children for outcomes of static/dynamic steady-state and proactive balance. Therefore, we conclude that balance performance improves from childhood up to late adolescence or early adulthood due to neural maturation and that balance might be differentially trainable in youth. Regarding sex differences, our analyses revealed inconsistent results, highlighting the need for well-designed, large cohort studies on this topic. Because our study was limited to the factors age and sex, further research is needed to determine differences in balance performance by additional factors (e.g., growth, maturation, expertise level).

## Supporting information

S1 Table Quality assessment of included studies. (XLSX)

S2 Table PRISMA-checklist–transparent reporting of systematic reviews and meta-analyses. (DOC)
